# Antibiotics Significantly Decrease the Survival of Head and Neck Carcinoma Patients with Immunotherapy: A Real-World Analysis of More Than 3000 Cases

**DOI:** 10.3390/cancers15082342

**Published:** 2023-04-18

**Authors:** Saskia Preissner, Max Heiland, Robert Preissner, Markus Wirth, Barbara Wollenberg

**Affiliations:** 1 Department of Oral and Maxillofacial Surgery, Charité—Universitätsmedizin Berlin, Augustenburger Platz 1, 13353 Berlin, Germany; 2 Institute of Physiology and Science-IT, Charité—Universitätsmedizin Berlin, Philippstr. 12, 10115 Berlin, Germany; 3 Clinic for Otorhinolaryngology, Head and Neck Surgery, Klinikum Rechts der Isar der Technischen Universität München (MRI TUM), Ismaningerstr. 22, 81675 Munich, Germany

**Keywords:** oral squamous cell carcinoma, oral cancer, immunotherapy, pembrolizumab, antibiotics, gut microbiome, survival rate, real-world data

## Abstract

**Simple Summary:**

It is well known that antibiotics alter the gut microbiome, and because it plays a role in drug metabolism, alterations to the microbiome may lead to ineffective immunotherapy in cancer patients. We investigated a real-world cohort of oral cancer patients who received immunotherapy. Patients were matched for age, sex, BMI, metastases, alcohol and nicotine dependence and sepsis to create two comparable groups. Patients who received antibiotics had a significantly decreased survival compared to those who did not. We believe that this finding is associated with less effective immunotherapy due to antibiotic-related changes in the gut microbiome.

**Abstract:**

Objective: The human gut microbiome is strongly influenced by the administration of drugs, namely antibiotics. We hypothesized that the effectiveness of immunotherapy with pembrolizumab in oral squamous cell carcinoma patients is decreased by the administration of antibiotics three months before and after immunotherapy. Methods: We retrieved data from patients diagnosed with head and neck squamous cell carcinoma (HNSCC) (International Classification of Diseases [ICD]-10 codes C00-C14) and receiving immunotherapy with pembrolizumab from the TriNetX network. Two cohorts were built: patients in cohort I did not receive any antibiotics within three months before or up to three months after immunotherapy, while patients in cohort II were administered antibiotics at least once within three months before or after immunotherapy. To exclude confounders, we matched cohorts 1:1 for age, sex, secondary lymph node metastases, nicotine dependence, the insertion of feeding devices, body mass index (BMI) and severe sepsis. After defining the primary outcome as “death”, a Kaplan–Meier analysis was performed, and the risk ratio (RR), odds ratio (OR) and hazard ratio (HR) were calculated. Results: A total of 3651 patients were enrolled, and after matching, each cohort consisted of 1362 patients. Among cohorts I and II, 346 and 511 patients were deceased within one year (risk of death = 25.5 and 38.3%, respectively), whereby the risk difference was significant (*p* = 0.000; log-rank test). The RR was 0.68 (95% confidence interval: 0.60–0.76), OR was 0.57 (0.48–0.67) and HR was 0.58 (0.51–0.67). Conclusions: Our hypothesis was confirmed: administering antibiotics significantly decreases the drug effectiveness of immunotherapy. We hypothesize that this finding is associated with antibiotic-related changes in the gut microbiome. Prospective clinical studies on the gut microbiome in cancer patients are necessary to understand the complex ecosystem of microbiota during immunotherapy. Trial Registration: Due to the retrospective nature of the study, no registration was necessary.

## 1. Introduction

Head and neck squamous cell carcinoma (HNSCC), among the most common cancers [1], is mainly caused by risk factors such as smoking and alcohol consumption. Despite intensive efforts, this cancer not been significantly reduced in recent decades. The current primary treatment regimen consists of surgery or radiation with or without chemotherapy [2]. In the setting of first-line treatment failure, the administration of PD-1 to target immune checkpoint inhibitors (ICIs), such as pembrolizumab (with chemotherapy or as a monotherapy) [3,4] or nivolumab [5], is well established and has become an integral part of treatment guidelines [6,7]. The benefit of these new agents is a reduction in severe treatment side effects, commonly associated with an extreme chemotherapy/Erbitux regimen, which leads to a massively improved quality of life, as well as significantly increased survival rates. Depending on the combined positive score (CPS) that reflects PD-L1 expression on tumor cells, infiltrating lymphocytes and macrophages, overall survival (OS) was significantly prolonged in patients receiving pembrolizumab compared to those receiving cetuximab–chemotherapy: PD-L1 CPS ≥ 20 and CPS ≥ 1, respectively. Pembrolizumab alone and pembrolizumab–chemotherapy also demonstrated a substantially longer duration of response (DOR) in all populations [8]. Similar effects can be observed in real-world treatment data on nivolumab monotherapy [9]. When comparing all ICI response rates in squamous cell cancers, it is striking that recurrent/metastatic head and neck cancers (R/M HNSCCs) are found within the lowest third of all cancers and have a response rate of 15–20%. To date, there is no obvious molecular or immunological reason why this response rate to ICI in R/M HNSCC is so low [10].

One possible explanation could be the timepoint of ICI treatment, which, to date, has only been available for patients in first-line failure settings such as recurrences or metastases. It is well-recognized that recurrent/metastatic cancers do not share clonal similarities with primary cancers in HNSCC [11,12]. New neoadjuvant treatment studies are already revealing different response rates to ICI [13,14,15].

Another reason could be the specifically altered microbiome of HNSCC patients. Very early on in the checkpoint treatment, several groups published data which found that primary resistance to ICIs can be attributed to abnormal gut microbiome composition, thereby [16,17] unravelling the importance of the commensal microbiota in immuno-oncology. Meanwhile, it is well known that this complex ecosystem is influenced by various drugs and can also influence treatment response [18,19]. In several pre-clinical models, the absence of an intact gut microbiome adversely affected ICI efficacy. So far in HNSCC, it remains unclear whether antibiotic-induced dysbiosis influences the clinical response through the modulation of the gut microbiome or whether it constitutes an additional surrogate marker of unfit or immunodeficient patients [20,21].

To investigate the influence of antibiotics on the clinical outcome of pembrolizumab treatment in a large real-world cohort, we selected the TriNetX Global Health Research Network (TriNetX, Cambridge, MA, USA) to take a closer look at patients’ data. The TriNetX provides access to a significant number of medical records from more than 78 healthcare organizations (HCOs) in 11 countries. Its intent is to bring together HCOs, contract research institutes and pharmaceutical companies to collect and exchange longitudinal clinical data and provide state-of-the-art statistical analytics. As of April 2023, TriNetX had collated electronic medical records of more than 250 million individuals. The network had previously been used to research medical topics of global importance, including the COVID-19 pandemic [22]. Detailed data analyses were performed to improve understanding of how the use of antibiotics before or after ICI therapy might change clinical prognoses.

## 2. Patients and Methods

### 2.1. Data Acquisition, Allocation and Matching

We retrieved data from patients diagnosed with head and neck squamous cell carcinoma (HNSCC) (International Classification of Diseases [ICD]-10 codes C00-C14) and receiving immunotherapy with pembrolizumab from the TriNetX network. Two cohorts were built: patients in cohort I did not receive any antibiotics within three months before or up to three months after immunotherapy, while patients in cohort II were administered antibiotics at least once within three months before or after immunotherapy. To exclude confounders, we matched cohorts 1:1 for age, sex, secondary lymph node metastases, nicotine dependence, insertion of feeding devices and body mass index (BMI).

### 2.2. Data Analysis

After defining the primary outcome as “death”, the time window was set to one year after the index event (ICD-10 codes C00-C14). Outcome events were recorded on a daily interval. Subsequently, a Kaplan–Meier analysis was performed, and the risk ratio (RR), odds ratio (OR) and hazard ratio (HR) were calculated. Statistical analysis was performed using the log-rank test, where *p* ≤ 0.05 was defined as significant. For 1:1 matching, a propensity score-matching algorithm was used. The system generated propensity scores for each patient in each cohort using logistic regression (software package scikit-learn). The propensity score ranged between 0 and 1 and indicated the predicted probability that a patient was in cohort I or II given the patient’s covariates. The greedy nearest-neighbor matching algorithm with a caliper of 0.1 pooled standard deviation was used. The caliper of 0.1 means that patients with very different propensity scores are not matched.

Furthermore, treatment pathways for the antibiotics used in cohort II were investigated.

## 3. Results

### 3.1. Assessment, Allocation and Matching

The access date was 6 April 2023, so no patients had to be excluded for index events that were more than 20 years old. A total of 42 HCOs responded with patients, and 3651 patients were enrolled. After matching for age, sex, secondary lymph node metastases, nicotine dependence, insertion of feeding devices, body mass index (BMI) and severe sepsis, each cohort accounted for 1362 patients. After matching, the cohorts did not differ significantly from each other (*p* > 0.05), as illustrated in Table 1.

**Figure 1:**
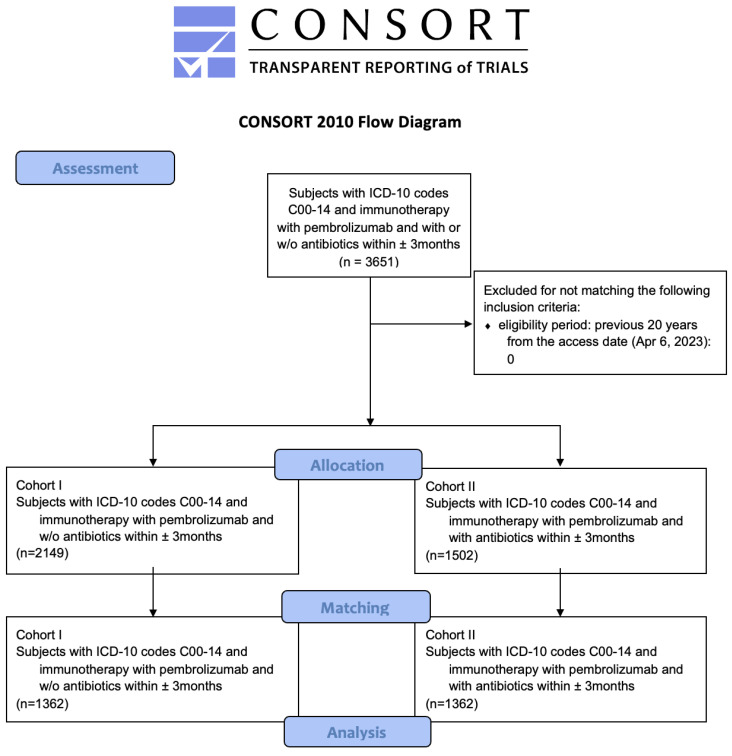
Modified Consort flow diagram. ICD-10: International Classification of Diseases 10, C00-14: malignant neoplasms of lip, oral cavity and pharynx.

Details about the assessment, allocation and matching can be found in the modified Consort diagram (Figure 1), as well as in Table 2.

### 3.2. Survival Analysis

In cohorts I and II, 346 and 511 patients, respectively, died within one year (risk of death = 25.4 and 37.5%), for which the risk difference was significant (*p* < 0.0001; log-rank test). The RR was 0.68 (95% confidence interval (CI) 0.60–0.76), OR was 0.57 (CI, 0.48–0.67) and HR was 0.58 (0.51–0.67).

Table 3 includes patients in each cohort with the outcome (death in the cohort and the number of patients that had the outcome in the time window), median survival (the number of days when the survival dropped below 50%; “--” indicates that survival did not drop below 50% during the time window of one year) and survival probability at the end of the time window (% survival at the end of the time window). In addition, a log-rank test, hazard ratio and *z*-test for proportionality were performed.

### 3.3. Treatment Pathways

**Figure 2:**
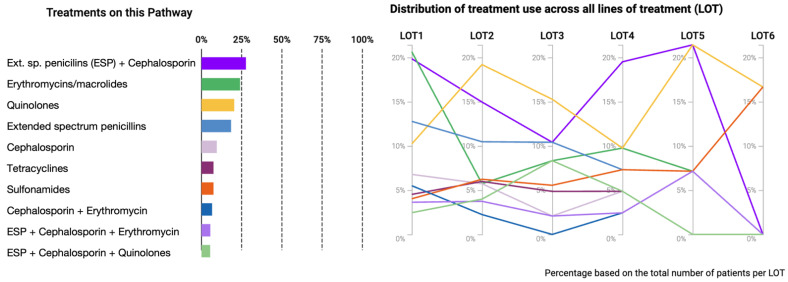
Treatment pathways for cohort 2 (with antibiotics). The left side shows the ten most administered treatments, and the right side shows six lines of treatment (LOT). Extended spectrum penicillins (ESPs).

For cohort II, a treatment pathway analysis was run. In the first line of treatment (LOT 1), the most administered regimen was erythromycin, followed by a combination therapy of extended spectrum penicillins and cephalosporins (third generation). Quinolones are more present in the second line of treatment (Figure 2).

## 4. Discussion

In concordance with many publications in recent years, our data confirmed that there is a crucial balance between human health and disease mediated by the gut microbiome, which modulates the host immune system both locally and systemically [23,24].

Especially in the context of immunotherapy, the gut microbiome appears to be among the most dominant biological markers for distinguishing therapy-responding patients from non-responders in various types of immunotherapies [25,26,27,28,29,30].

Although not understood in mechanistic details, emerging evidence indicates that disruption of the intestinal flora by different antibiotics can result in several negative consequences for the gut microbiota: the reduced diversity of species and an alteration in metabolic activity and the selection of antibiotic-resistant organisms [31,32]. This fact is not only important during the early assembly of the gut microbiome during neonatal growth [33] but especially during cancer therapy, such as surgery in non-sterile areas such as the head and neck region or during radiotherapy [34,35] and in immunotherapy especially [36]. The sample size of those studies reviewed in meta-analyses is usually low, which motivated the authors of our study to analyze a large database of malignant neoplasms of the head and neck. In total, 1502 datasets were available for patients with antibiotics, and 2149 datasets were available for those without antibiotics during or before immunotherapy. By exploiting the TriNetX Global Health Research Network, we can prove that the drug effectiveness of immunotherapy is decreased by the administration of antibiotics. Individuals who were administered antibiotics within ±3 months of immunotherapy had a significantly higher risk of dying within one year compared to individuals who were not administered antibiotics.

There are studies analyzing the degree of perturbation of the commensal microbiome by four commonly used antibiotics: azithromycin, levofloxacin, cefpodoxime and combinations thereof [37]. Consequently, if antibiotic therapy cannot be avoided in immunotherapy, certain drug regimens such as azithromycin and its combinations should be avoided as they have been shown to delay for two months the recovery of species richness. Whether probiotic administration or an additional immunotherapy delay improves the outcome of anti-cancer immunotherapy needs to be examined in prospective clinical trials. Furthermore, it is unclear whether the parameters used to characterize the gut microbiome, such as species richness or aerobic/anaerobic composition, are relevant to the differences in the observed outcomes. Mechanistic studies could help identify certain relevant species, (biosynthetic) pathways, messengers, metabolites or other supportive effects of the microbiome.

Alongside the applied methods are specific limitations regarding the results that need to be addressed. Most importantly, we had no data on the microbiome of the patients, so we used the administration of antibiotics as a marker for an altered gut microbiome [38]. By matching the cohorts for age, sex, lymph node metastases, BMI, feeding device, smoking habits and severe sepsis, we designed two cohorts that were as similar as possible. A disease-specific survival analysis would be interesting, but due to the de-identified data, we had no data on individual causes of death. It must be mentioned that tobacco use is self-reported, and we had no detailed data on tobacco use (e.g., pack-years) [39]. A certain risk of confounders, such as metabolic alterations after alcohol consumption, an important lifestyle factor frequently associated with smoking, has yet to be comprehensively investigated, so a certain bias should be considered [40]. Another important limitation was the lack of information on the form of antibiotic administration: oral or intravenous. Depending on how the drug was metabolized, agents with no or low bile or fecal excretion were available and may have affected the gut microbiome less. The implications of drug excretion on the gut microbiome are still controversial [41].

Future studies may apply a prospective approach to include data, especially on the microbiome before, during and after treatment, and smoking/drinking behavior. Furthermore, tumor status (e.g., HPV+) should include lifestyle factors and the form of drug administration. Particularly in HNSCC reconstructive surgery, long phases of parental tube feeding might add an additional load to gut dysbiosis, so special care should be applied with regard to the length and composition of the applied nutrients.

## 5. Conclusions

In summary, our data impressively underlined the assumptions of studies connecting antibiotics-induced dysbiosis and therapeutic outcomes. At the very least, the harvested data support our hypotheses of decreased therapy effectiveness due to antibiotic use. This might encourage further research on the gut and locoregional microbiome in head and neck cancer patients. If the results could be confirmed in the future, the microbiome should be considered in individualized cancer therapy, e.g., for applying supportive probiotics when antibiotics must be administered [42].

## Figures and Tables

**Table 1:** Propensity score density function (purple: cohort I, green: cohort II).

Propensity Score Density Function—Before (Left) and after (Right) Matching (Cohort I—Purple, Cohort II—Green)	
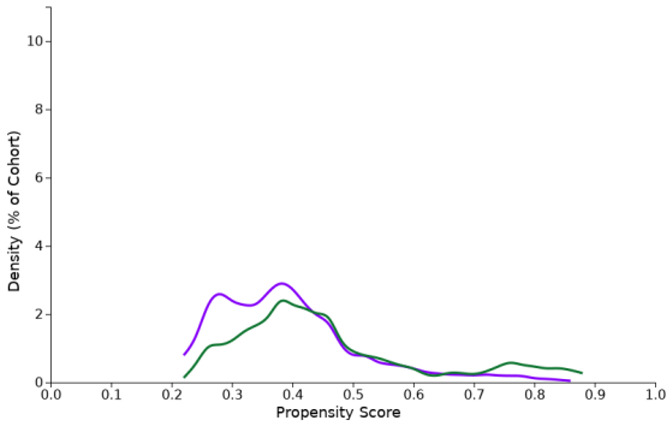	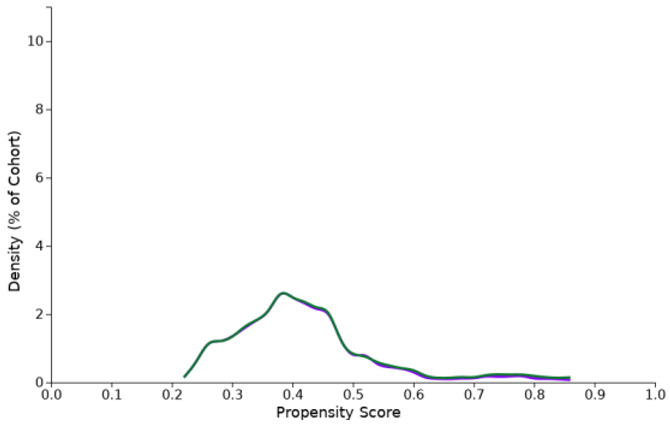

**Table 2:** Characteristics of cohorts I (individuals with ICD-10 codes C00-14 and no antibiotics) and II (with antibiotics) before and after matching for age and sex, BMI, secondary lymph node metastases, tobacco use and feeding device. ICD-10 = International Classification of Diseases.

	Before Matching				After Matching			
Patients (n)	Cohort I	Cohort II	*p*-Value	Standardized Mean Difference	Cohort I	Cohort II	*p*-Value	Standardized Mean Difference
Total	2149	1502			1362	1362		
Age at index	65.7	64.0	<0.001	0.141	64.5	64.4	0.773	0.011
Standard deviation	12.1	12.2			12.1	12.1		
Males	1579 73.5%	1117 74.4%	0.546	0.020	1043 76.6%	1007 73.9%	0.110	0.061
Females	570 26.5%	385 25.6%	0.546	0.020	319 23.4%	355 26.1%	0.110	0.061
BMI	24.8	23.9	<0.001	0.148	24.6	24.1	0.065	0.086
Standard deviation	5.6	5.4			5.4	5.4		
Metastases	1126 31.8%	890 40.1%	<0.001	0.138	785 57.6%	788 57.9%	0.907	0.004
Tobacco use	684 32.1%	603 35.4%	0.059	0.070	524 38.5%	523 38.4%	0.969	0.002
Feeding device	363 16.9%	423 28.1%	<0.001	0.138	319 23.5%	322 23.6%	0.961	0.002
Severe sepsis	41 1.9%	145 9.7%	<0.001	0.337	40 2.9%	43 3.2%	0.738	0.013

**Table 3:** Kaplan–Meier survival analysis for one year (purple: cohort I, green: cohort II).

Cohort		Patients in Cohort	Patients with Outcome	Median Survival (days)	Survival Probability at End of Time Window	
I	w/o antibiotics	1362	346	--	68.42%	
II	w antibiotics	1362	511	--	53.89%	
		χ^2^	df	*p*		
Log-rank test		61.522	1	0.000		
		Hazard Ratio	95% CI	χ^2^	df	*p*
Hazard ratio and proportionality		0.58	(0.51, 0.67)	6.52	1	0.011
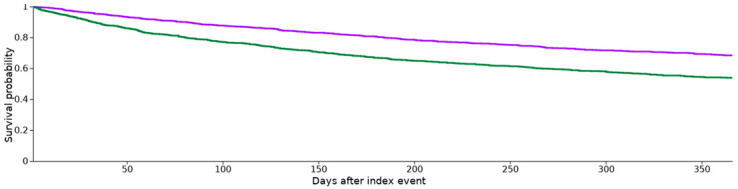						

## Data Availability

The datasets used and analyzed can be retrieved from the TriNetX network (https://trinetx.com, accessed on 5 May 2022). If no access is available, the datasets can be retrieved from the corresponding author based on reasonable request.

## Abbreviations

HCO	heath care organization
HPV	human papillomavirus
ICD-10	International Classification of Diseases 10
OR	odds ratio
HR	hazard ratio
HNSCC	head and neck squamous cell carcinoma
RR	risk ratio
UICC	Union Internationale Contre le Cancer
WHO	World Health Organization
